# A Dominant-Negative PPAR*γ* Mutant Promotes Cell Cycle Progression and Cell Growth in Vascular Smooth Muscle Cells

**DOI:** 10.1155/2009/438673

**Published:** 2010-03-10

**Authors:** Joey Z. Liu, Christopher J. Lyon, Willa A. Hsueh, Ronald E. Law

**Affiliations:** ^1^The Methodist Hospital Research Institute, Houston, TX 77030, USA; ^2^Takeda America Holdings, Deerfield, IL 60015, USA

## Abstract

PPAR*γ* ligands have been shown to have antiproliferative effects on many cell types. We herein report that a synthetic dominant-negative (DN) PPAR*γ* mutant functions like a growth factor to promote cell cycle progression and cell proliferation in human coronary artery smooth muscle cells (CASMCs). In quiescent CASMCs, adenovirus-expressed DN-PPAR*γ* promoted G1→S cell cycle progression, enhanced BrdU incorporation, and increased cell proliferation. DN-PPAR*γ* expression also markedly enhanced positive regulators of the cell cycle, increasing Rb and CDC2 phosphorylation and the expression of cyclin A, B1, D1, and MCM7. Conversely, overexpression of wild-type (WT) or constitutively-active (CA) PPAR*γ* inhibited cell cycle progression and the activity and expression of positive regulators of the cell cycle. DN-PPAR*γ* expression, however, did not up-regulate positive cell cycle regulators in PPAR*γ*-deficient cells, strongly suggesting that DN-PPAR*γ* effects on cell cycle result from blocking the function of endogenous wild-type PPAR*γ*. DN-PPAR*γ* expression enhanced phosphorylation of ERK MAPKs. Furthermore, the ERK specific-inhibitor PD98059 blocked DN-PPAR*γ*-induced phosphorylation of Rb and expression of cyclin A and MCM7. Our data thus suggest that DN-PPAR*γ* promotes cell cycle progression and cell growth in CASMCs by modulating fundamental cell cycle regulatory proteins and MAPK mitogenic signaling pathways in vascular smooth muscle cells (VSMCs).

## 1. Introduction

VSMC proliferation is an early response to the arterial wall injury, as well as the primary event in more extensive vascular remodeling associated with increased intima-media thickness and atherosclerotic lesions [1]. Both damaged and activated VSMCs secrete growth factors and cytokines that trigger multiple mitogenic signaling pathways, including the Ras/Raf/MEK/ERK signaling cascade [2]. ERK activation induces cyclin D1 expression and thus facilitates the formation of the cyclinD1-CDK4/6 complex, the key step for quiescent cells to undergo cell cycle entry [3]. Active cyclinD1-CDK4/6 complexes phosphorylate retinoblastoma protein (Rb), releasing sequestered E2F bound to hypophosphorylated Rb to promote transcription of key cell cycle genes required for S phase DNA replication [4]. E2F release induces the expression of regulatory proteins involved in the initiation step of chromosomal DNA replication, such as the minichromosome maintenance (MCM) proteins, which are recruited to replication origins during the G1 phase of the cell cycle, establishing the competence of these origins for initiation of DNA replication in the subsequent S phase [5]. 

Several groups, including our own, have shown that PPAR*γ* ligands, such as the thiazolidinediones (TZDs), inhibit VSMC proliferation and cell cycle progression in vitro [6–11] and intimal hyperplasia in vivo [7]. Despite extensive study, however, the mechanism(s) underlying the antiproliferative effect of PPAR*γ* ligands in VSMC remain to be determined. Rare and natural PPAR*γ* mutations have been associated with insulin resistance, hypertension, and vascular hypertrophy [12–14], with the majority of mutations reported to date found in the ligand-binding domain (LBD) of the receptor [15]. Gurnell et al. [16] created an artificial PPAR*γ* LBD mutant by introducing L486A and E471A amino acid substitutions. This mutant exhibited dominant-negative activity, suppressing the activity of cotransfected WT PPAR*γ* and blocking TZD-induced adipogenesis in 3T3-L1 preadipocytes. The profound dominant-negative effects of this mutant were attributed to impaired release of corepressors (NCoR and SMRT) and diminished recruitment of coactivators (CBP and SRC). A DN-PPAR*γ* construct has been shown to promote neointima formation in balloon-injured rat arteries and enhance VSMC proliferation and migration [17]. They reported that injury-induced intima-media ratio (IMR) was reduced in animals infected with adenovirus expressing WT PPAR*γ*; however, rats infected with adenovirus expressing DN-PPAR*γ* demonstrated significantly greater IMRs than untreated controls, regardless of PPAR*γ* ligand treatment. Recently, Meredith et al. [18] demonstrated that VSMC isolated from transgenic mice harboring a dominant-negative mutation of PPAR*γ* showed greater proliferation and migration compared to VSMC isolated from wild-type mice.

Based on these studies, we hypothesized that a DN-PPAR*γ* mutant would antagonize WT-PPAR*γ* activity to abrogate or reverse its effects on VSMC cell growth. We therefore examined the effect of DN-PPAR*γ* expression on cell cycle progression, G1 to G2/M cell cycle regulators, and MAPK mitogenic signaling pathways in VSMCs and found that DN-PPAR*γ* promoted the expression and activity of positive regulators of the cell cycle, G1→S progression and cell proliferation, and that these effects were mediated in part through ERK activation. 

## 2. Materials and Methods

### 2.1. Cell Culture

Primary human CASMCs and SmGM-2 and SmBM cell culture media were purchased from Cambrex (Walkersville, MD). Early passage (four to nine) CASMCs were cultured to ~75% confluence in SmGM-2 growth medium, then cultured from 24 to 48 hours in SmBM basal medium supplemented with 0.4% FBS to induce cell cycle arrest. Mouse PPAR*γ*-deficient embryonic stem (ES) cells were kindly provided by Dr. R. Evans (Howard Hughes Med. Ins., Lo Jolla, CA) and described previously [19]. Murine primary embryonic fibroblasts used as ES feeder cells were purchased from Stem Cell Technologies (Vancouver, BC, Canada). ES cells were maintained and differentiated with ES-Cult products (Stem Cell Technologies) according to the manufacturer's protocols.

### 2.2. Adenovirus Procedures

An expression vector containing full-length human wild-type PPAR*γ*1 [20] was kindly provided by Dr. Alex Elbrecht (Merck Research Laboratories, Whitehouse Station, NJ), and used to create the DN-PPAR*γ* double-mutant described by Gurnell et al. [16] by mutations of L468A and E471A using a QuikChange Multi Site-Directed Mutagenesis Kit (Stratagene, La Jolla, CA). An expression vector containing a constitutively-active mouse PPAR*γ*1 (CA-PPAR*γ*) mutant, created by fusing the herpes simplex VP16 transactivation domain to the N-terminus of mouse WT PPAR*γ*1 [21], was a kind gift from Dr. Barry Forman (City of Hope National Medical Center, Duarte, CA). An expression vector encoding the VP16 transactivation domain only (pTet/VP16) was purchased from Clontech. Adenovirus expressing WT-PPAR*γ* (Ad-WT), DN-PPAR*γ* (Ad-DN), CA-PPAR*γ* (Ad-CA), the VP16 transactivation domain (Ad-VP16), and the green fluorescent protein (Ad-GFP) were created with the Adeno-X Expression System (Clontech, Mountain View, CA) according to the manufacturer's protocol, confirmed by DNA sequencing, then expanded in HEK293 cells and purified with Adeno-X Virus Purification Kits (Clontech). Viral titers were determined with Clontech Adeno-X Rapid Titer Kit, and virus was used at multiplicity-of-infection (MOI) rates of 20 to 80 infectious particles per cell, as indicated. CASMCs were growth-arrested by culture in SmBM basal medium with 0.4% of FBS for 24 to 48 hours, as indicated, before 48 hours infection with adenovirus. Specified cultures were supplemented with or without serum, growth factors, or the ERK MAPK inhibitor PD98059 (Cell Signaling Technology, Danvers, MA) at the indicated times and conditions.

### 2.3. Luciferase Assays

NIH/3T3 cells (American Type Culture Collection, Manassas, VA) were transfected for 48 hours with 1 *μ*g DN-, WT-, or CA-PPAR*γ* plasmid DNA, and 5 ng of CMV-renilla luciferase (pRL-CMV; Promega, Madison, WI) and 1 *μ*g of acyl-CoA oxidase PPAR Response Element (PPRE, 3x)-tk-firefly luciferase DNA (kind gift of Dr. Peter Tontonoz, UCLA) using LipofectAMINE 2000 (Invitrogen, Rockville, MD) according to the manufacturer's protocol. After 24 hours transfection, cells were treated with 10 *μ*M rosiglitazone (RSG) in DMSO or DMSO alone. Luciferase activity was assayed 48 hours after transfection with the Dual Luciferase Reporter Assay System (Promega) following the manufacturer's instructions. PPRE-driven firefly luciferase activity was normalized to CMV-driven renilla luciferase activity to adjust for differences in transfection efficiency.

### 2.4. Cell Cycle Analysis

 After serum-starvation, CASMCs used for cell cycle analysis were infected with virus for 48 hours with or without PDGF plus insulin, and then resuspended by trypsinization, centrifuged for 5 minutes at 250 × g, washed with PBS, centrifuged for 5 minutes at 250 × g, and aspirated to remove supernatant from the cell pellet. CASMC cell pellets were then resuspended in DNA staining buffer (3.4 mM sodium acetate, 0.3% Triton X-100, 1.5 mM propidium iodide, and 20 *μ*g/mL RNase A) and incubated at 4°C for 30 minutes. Nuclei staining data was acquired by flow cytometry using a Becton Dickinson FACScan, and the proportion of cells in G0/G1, S, and G2/M phase were determined using Becton Dickinson ModFit LT software.

### 2.5. DNA Synthesis and Cell Proliferation Assays

CASMCs used for BrdU incorporation and cell proliferation assays were grown to ~75% confluence in 60-mm plates, serum-starved for 48 hours in SmBm basal medium containing 0.4% FBS, and infected for 48 hours with adenovirus expressing GFP, DN-, or CA-PPAR*γ*. Cells were then resuspended by trypsinization and replated in 96-well plates at a density of 1 × 10^4^ cells/well and cultured for 3 days in SmBm basal medium containing 0.4% FBS. BrdU incorporation analyses were then carried out using a commercially available BrdU immunoassay (Calbiochem, San Diego, CA) according to the manufacturer's instructions. Cells used for DNA proliferation analysis were infected with virus for 48 hours and then recultured in 12-well plates at 5 × 10^4^ cells/well for another 3 days in SmBm basal medium containing 0.4% FBS. Cells were then resuspended by trypsinization and counted with a hemocytometer.

### 2.6. Western Blot Analyses

CASMC whole cell lysates were generated as previously described [22], size fractionated by SDS-PAGE, and transferred onto Hybond ECL nitrocellulose membrane (Amersham Biosciences, Piscataway, NJ) prior to Western blot analysis. Specific antibodies for phospho-Rb (Ser807/811), phospho-cdc2 (Thr161), cyclin D1, phospho- and total ERK (p44/42), and horseradish peroxidase conjugated secondary antibodies were purchased from Cell Signaling Technology. Cyclin A (sc-751), MCM7 (sc-9966), p21 (sc-6246), p27 (sc-1641), and PPAR*γ* (sc-7196) specific antibodies were purchased from Santa Cruz Biotechnology. Specific antibodies to cyclin B1 (05-158; Millipore), GFP and *β*-actin (Abcam, Cambridge, MA), and VP16 (Clontech) were purchased from the indicated companies. Specific antibody hybridization was detected using ECL reagents and X-ray film (Amersham), and resulting signals were quantified by densitometry. Western blot protein expression was normalized against *β*-actin, except pERK1/2, which was normalized against total ERK1/2.

### 2.7. Statistical Analyses

Data are presented as mean ± standard error of the mean (SE). Differences between individual groups were analyzed by 2-tailed Student's *t*-test or 1-way analysis of variance (ANOVA) followed by Tukey-Kramer posttests to determine differences between individual means when comparing multiple groups. A *P*-value ≤.05 was considered statistically significant for all tests.

## 3. Results

### 3.1. DN-PPAR*γ* Expression Completely Inhibits WT-PPAR*γ* Activity

**Figure 1:**
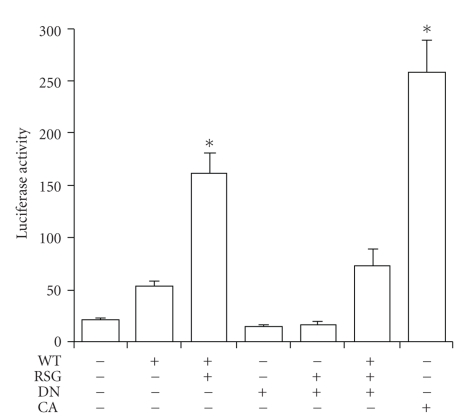
PPRE-mediated transcriptional activity of WT-, DN-, and CA-PPAR*γ*. NIH/3T3 fibroblasts were transiently transfected with a PPRE(3x) firefly luciferase reporter vector and a pRL-CMV renilla luciferase expression construct with or without plasmids expressing WT-, DN-, or CA-PPAR*γ*. After 24 hours of transfection, cells were treated with or without 10 *μ*M rosiglitazone (RSG). Luciferase activity was assayed 48 hours after transfection, adjusting for transfection efficiency by normalizing firefly luciferase activities to renilla luciferase activity. All transfections were performed a minimum of three times. Data are expressed as the ratios of firefly to renilla luciferase activity and presented as mean ± SE (*P < .05 versus all other conditions by 1-way ANOVA).

 NIH/3T3 fibroblasts, which do not express detectable PPAR*γ* [23], were transfected with a PPRE-luciferase reporter vector and plasmids expressing WT-, DN-, or CA-PPAR*γ*, or empty expression vector in the presence or absence of the PPAR*γ* ligand RSG (Figure 1). Ligand treatment increased luciferase activity 3-fold in cells transfected with WT-PPAR*γ* vector. Cells transfected with CA-PPAR*γ* vector revealed a greater than 13-fold increase in luciferase activity compared to those transfected with empty expression vector constructs. Transfection with DN-PPAR*γ*, with or without RSG, did not result in appreciable change of luciferase activity compared to the basal level. However, when cotransfected with WT-PPAR*γ*, the mutant PPAR*γ* repressed WT-PPAR*γ* transcriptional activity by 58% (Figure 1). These data agree with previously reported results [16], confirming that the L486A/E471A double-mutant PPAR*γ* behaves as a dominant-negative that fully suppresses wild-type PPAR*γ* activity.

### 3.2. DN-PPAR*γ* Promotes G1→S Progression and Cell Growth

**Figure 2:**
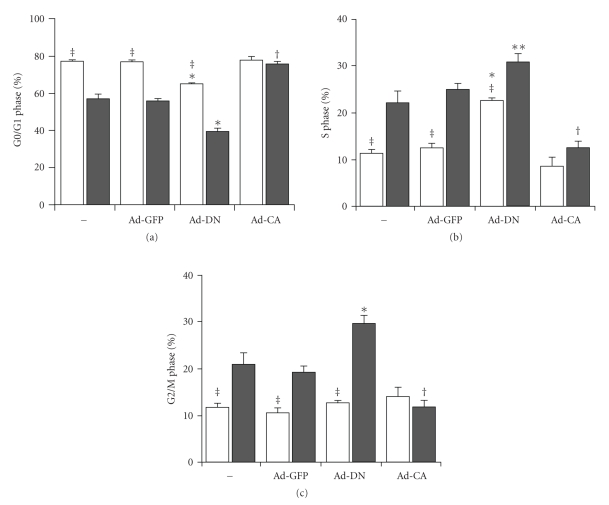
DN-PPAR*γ* promotes CASMC cell cycle G1→S progression. CASMCs were cultured in serum-depleted medium (0.4% FBS) for 48 hours to induce cell cycle arrest, then infected with or without (−) adenovirus expressing GFP, DN-PPAR*γ*, or CA-PPAR*γ* (MOI of 40). After 24 hours, cells were cultured with vehicle (white bars) or with 20 ng/mL PDGF plus 0.1 *μ*M insulin (grey bars) for 24 hours to induce cell proliferation and then harvested and stained with propidium iodide. The fractions of G0/G1, S, and G2/M phase cells in each sample were determined by measuring the DNA content of 1 × 10^6^ cells per sample by flow cytometry. Results presented represent the mean ± SE of at least four independent experiments. (*P < .05 versus no virus (−), Ad-GFP and Ad-CA; **P < .05 versus no virus and Ad-CA; and ^†^ *P* < .05 versus no virus, Ad-GFP and Ad-DN for the matching culture condition by 1-way ANOVA; ^‡^ *P* < .05 versus matching serum-stimulated culture by Student's *t*-test).

In order to assess the effect of DN-PPAR*γ* expression on cell-cycle progression, CASMCs were serum starved to induce cell cycle arrest at G0/G1, infected with Ad-GFP, Ad-CA, or Ad-DN, then treated with PDGF plus insulin to stimulate cell cycle progression. Flow cytometry analysis (Figure 2) showed that CASMCs infected with Ad-GFP had cell cycle profiles similar to noninfected cells at all stages of the cell cycle, both during growth arrest and stimulation. CASMCs infected with Ad-DN, however, demonstrated significant decreases in the number of cells in the G0/G1 phase of the cell cycle when compared to uninfected and Ad-GFP infected cells, both during growth arrest (65.0% versus 77.2% and 76.9%) and stimulation (39.4% versus 57.0% and 55.8%). Serum-starved CASMCs infected with Ad-DN demonstrated an increased percentage of S phase cells, relative to uninfected and Ad-GFP infected CASMCs (22.6% versus 11.3% and 12.5%), but revealed a similar percentage of cells in G2/M. Stimulation of these cells with PDGF and insulin increased the percentage of S phase and G2/M phase cells, but Ad-DN infected cells had a greater percentage of cells in both S and G2/M than any of the other samples. 

**Figure 3:**
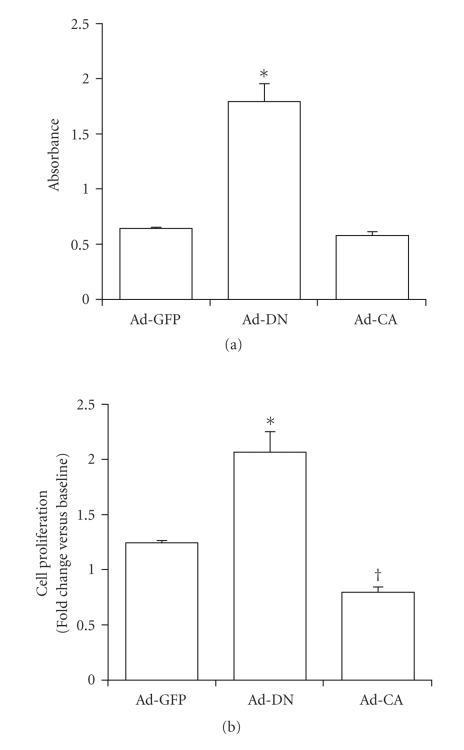
DN-PPAR*γ* stimulates DNA synthesis and cell proliferation of quiescent CASMCs. Cells were cultured in serum-depleted medium (0.4% FBS) for 48 hours to induce cell cycle arrest, then infected with Ad-GFP, -DN or -CA (MOI = 40) for 48 hours. (a) Cells were trypsinized, counted, and reseeded into 96-well plates at a density of 1 × 10^4^ cells/well. *N* = 3 wells/sample. After 3 days culture in serum-depleted medium, incorporation of the BrdU thymidine analog was assayed using a commercially available immunoassay (*P < .05 versus Ad-GFP control). (b) After infection with virus, cells were reseeded in 12-well plates at a density of 5 × 10^4^ cells/well and cultured in serum-depleted medium. After 3 days, cells were harvested and counted on a hemocytometer to measure cell proliferation. *N* = 4-5 counts/sample (*P < .05 versus Ad-GFP control; ^†^ *P* < .05 versus Ad-GFP control and Ad-DN).

Serum-starved CASMCs infected with Ad-CA were not significantly different from noninfected or Ad-GFP infected cells at any stage of the cell cycle. However, Ad-CA-infected CASMCs induced with PDGF and insulin demonstrated marked attenuation of cell cycle progression, demonstrating cell cycle profiles that appeared no different from those of growth arrested CASMCs. As shown in Figure 3(a), DNA synthesis measured by BrdU incorporation was significantly increased in Ad-DN-infected CASMCs (2.8-fold versus Ad-GFP), confirming the ability of DN-PPAR*γ* to stimulate G1→S progression under serum starvation conditions. These findings demonstrate that in CASMCs DN-PPAR*γ* expression promotes G1→S transition, and that CA-PPAR*γ* expression can completely block mitogen-stimulated cell cycle progression. This data agreed well with previous reports that indicate that PPAR*γ* ligands can suppress cell cycle progression, since dominant-negative suppression of PPAR*γ* activity promoted G1→S progression in both quiescent and simulated CASMCs, while constitutively-active PPAR*γ* did not appear to affect the G1→S phase transition in quiescent cells but completely attenuated the G1→S phase increase normally observed upon growth stimulation with PDGF and insulin. 

Since DN-PPAR*γ* stimulated G1→S cell cycle progression and DNA synthesis, even in the absence of exogenous mitogens, we next examined the ability of DN-PPAR*γ* to promote cell cycle progression through G2/M to promote CASMC proliferation. As shown in Figure 3(b), after 3-day culture under serum-starvation, Ad-DN-infected CASMCs demonstrated a significant increase in cell number when compared to Ad-GFP-infected cells (2.1-fold versus 1.2-fold starting cell number, resp.). Conversely, Ad-CA-infected CASMCs revealed a significant decrease in cell number (0.8-fold starting cell number), which may be explained by our previous data indicating that PPAR*γ* ligands can induce VSMC apoptosis [22].

### 3.3. DN-PPAR*γ* Induces the Expression of Key Regulatory Cell Cycle Proteins

Since the induction of cell cycle progression and cell growth by DN-PPAR*γ* expression likely resulted from changes in cell cycle checkpoint proteins, we examined the expression of key regulatory proteins required for progression through the G0/G1, S, and G2/M cell cycle checkpoints and the expression of two cyclin-dependent kinase inhibitors (CDKIs) known to regulate the activity of several checkpoint proteins.

**Figure 4:**
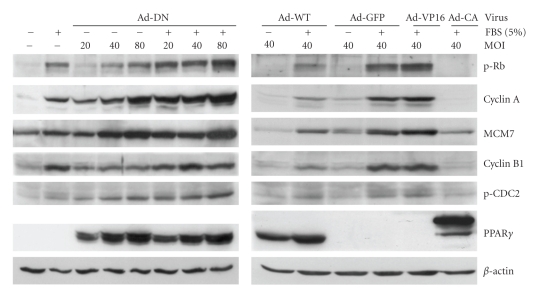
Ad-DN up-regulates, while Ad-WT and Ad-CA down-regulate, positive regulators of G0/G1, S, and G2/M phases. CASMCs were arrested in serum-deprived medium for 24 hours, then infected with or without Ad-DN, -WT, -GFP, -VP16, or -CA recombinant adenoviruses at the indicated MOIs for 48 hours, with or without serum stimulation. Western blot analyses were performed on whole cell extracts of these samples using antibodies against the indicated cell cycle regulatory proteins, then stripped and rehybridized with a *β*-actin specific antibody to assess loading variability. Western blots shown are representative of at least four independent experiments.

 As shown in Figure 4, CASMCs infected with Ad-DN strikingly upregulated the activity of several genes required at cell cycle checkpoints, increasing Rb phosphorylation (G1→S), cyclin A expression (S→G2 and G2→M), MCM7 expression (S phase DNA synthesis), and cyclin B1 expression/CDC2 phosphorylation (G2→M), and these effects were predominantly dose-dependent. For example, in serum-starved CASMCs, Ad-DN, at MOI 40, increased cyclin B1 (2-fold), p-CDC2 and MCM7 (4-fold), pRb (8-fold) and cyclin A (19-fold) versus serum-starved, uninfected cells. Serum induced similar increases in the expression of these proteins (cyclin B1, p-CDC2 and MCM7 3- to 4-fold, pRb 14-fold, and cyclin A 20-fold). Comparable results were observed when 20 ng/mL PDGF plus 0.1 *μ*M insulin was used to stimulate cells proliferation instead of 5% FBS (data not shown). Ad-GFP had no effect on basal or serum-stimulated expression of these proteins, while in the absence of serum stimulation Ad-WT suppressed only MCM7 expression. Ad-DN dose-dependently increased serum-induced cell cycle protein expression. Ad-DN infection at 40 MOI only increased cyclin A (2-fold), while Ad-DN at 80 MOI increased p-CDC2, pRb, and cyclin A expression (2- to 3-fold), under serum-stimulation condition. By contrast, Ad-WT and Ad-CA suppressed serum-induced increases in pRb, cyclin A, cyclin B1 and MCM7, but not p-CDC2. Ad-WT decreased cyclin A and B1, MCM7 and pRb expression to 30%–50% of serum-induced CASMC expression in the absence of exogenous ligand. Ad-CA markedly reduced pRb and cyclin A (2%-3% of serum control), cyclin B1 (12% of control), and MCM7 (30% of control), demonstrating that activated PPAR*γ* strongly reduced the expression or activity of these regulatory cell cycle proteins.

**Figure 5:**
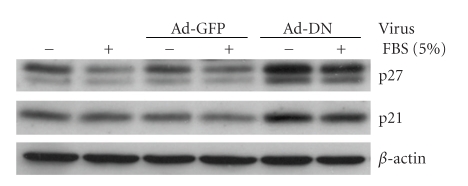
Ad-DN expression up-regulates CASMC expression of CDKIs p21 and p27. CASMCs were arrested in serum-deprived medium for 24 hours, then infected with or without Ad-GFP or -DN recombinant adenoviruses (MOI = 80) for 48 hours, with or without serum stimulation. Western blot analyses were performed on whole cell extracts of these samples using antibodies specific for CDKIs p21 and p27, then stripped and rehybridized with a *β*-actin specific antibody to assess loading variability. Western blots shown are representative of at least three independent experiments.

Surprisingly, Ad-DN infection also increased the protein level of two important negative regulatory CDKIs, p21 and p27 (Figure 5), by approximately 2-fold versus either uninfected or Ad-GFP infected samples. We did not observe any Ad-DN effect on CDKI p16 (data not shown). These results suggest that DN-PPAR*γ* expression regulates both positive and negative regulators of cell cycle progression.

### 3.4. DN-PPAR*γ* Effect on Cell Cycle Is PPAR*γ* Dependent

**Figure 6:**
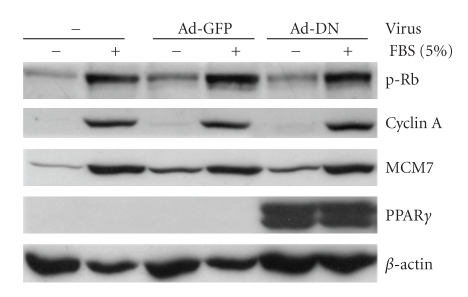
DN-PPAR*γ* does not up-regulate phospho-Rb, cyclin A, or MCM7 in PPAR*γ*-deficient embryonic stem cells. PPAR*γ* ^−/−^ ES cells were differentiated and then growth arrested in serum-deprived medium for 24 hours, then infected with or without Ad-GFP, -DN, or WT recombinant adenovirus (MOI = 80) for 48 hours, with or without serum stimulation. Western blot analyses were performed on whole cell extracts of these samples using antibodies specific for the indicated regulatory cell cycle proteins, then stripped and rehybridized with a *β*-actin specific antibody to assess loading variability. Western blots shown are representative of at least three independent experiments.

In order to confirm the role of PPAR*γ* in the regulation of cell cycle proteins, we examined the effects of DN-PPAR*γ* and GFP expression on the expression of regulatory cell cycle proteins in mouse PPAR*γ* ^−/−^ embryonic stem (ES) cells [19]. Again, PPAR*γ* ^−/−^ ES cells demonstrated marked induction of cyclin A, MCM7, and pRb expression upon serum stimulation (Figure 6). However, unlike our previous results with Ad-DN-infected CASMCs, Ad-DN infection of PPAR*γ* ^−/−^ ES did not induce cyclin A, MCM7, or pRb expression versus Ad-GFP-infected cells under either serum-starvation or -stimulation conditions. This did not appear to result from differential PPAR*γ* responses in ES versus CASMC, since Ad-DN induction of cyclin A, MCM7, and pRb expression in WT ES cells was similar to that observed in CASMCs (data not shown). These data strongly suggest that DN-PPAR*γ* exerts its growth promoting effects through attenuation of an endogenous PPAR*γ* activity to suppress cell proliferation.

### 3.5. DN-PPAR*γ* Activates ERKs 1/2 to Induce Expression of Cell Cycle Proteins

**Figure 7:**
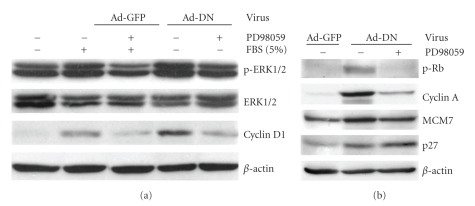
DN-PPAR*γ*-mediated induction of cell cycle regulators is ERK MAPK-dependent. (a) Ad-DN induced phosphorylation of ERK1/2 and expression of cyclin D1, which are inhibited by the ERK MAPK inhibitor PD98059. (b) PD98059 inhibits DN-PPAR*γ*-induced upregulation of phospho-Rb, cyclin A, and MCM7 but increases p27 expression. CASMCs were arrested in serum-deprived medium for 48 hours, cultured with 30 *μ*M PD98059 for 30 minutes prior to infection with or without Ad-GFP or -DN recombinant virus for 48 hours, in the absence of serum. Western blot analyses were performed on whole cell extracts of these samples using antibodies specific for the indicated proteins, then stripped and rehybridized with a *β*-actin specific antibody to assess loading variability. Western blots shown are representative of at least three independent experiments.

Serum or mitogenic growth factors induce phosphorylation of the mitogen activated protein kinases ERK 1 and ERK 2, activating signal transduction cascades that promote cell growth. Activated ERK 1/2 regulate the cell cycle, in part, by transducing signals to the nucleus that leads to increased expression of cyclin D1, a key protein in the regulation of the G1 to S phase transition [2, 24]. Serum and Ad-DN similarly induced CASMC ERK1/2 phosphorylation (2- and 3-fold, resp.) and cyclin D1 protein expression (10- and 19-fold, resp.), both of which were attenuated by the ERK specific-inhibitor PD98059 (Figure 7(a)). Similarly, treatment of CASMCs with PD98059 also attenuated DN-PPAR*γ*-mediated induction of several positive cell cycle regulators (Figure 7(b)), suggesting that the previously observed cell cycle and growth effects of DN-PPAR*γ* were mediated, at least in part, through activation of the ERK1/2 signaling pathway. Moreover, PD98059 treatment markedly attenuated DN-PPAR*γ*-mediated Rb phosphorylation and cyclin A and MCM7 expression, but increased p27 expression, suggesting that DN-PPAR*γ* stimulates the expression of positive and negative regulators of the cell cycle via different mechanisms.

## 4. Discussion

In the present study, we demonstrate that a dominant-negative PPAR*γ* mutant harboring L486A/E471A substitutions in its LBD is a potent activator of cell cycle progression and cell proliferation in quiescent (serum-starved) human CASMCs. We found that Rb and CDC2 phosphorylation and expression of cyclins A and B1, and MCM7 were strongly induced after quiescent CASMCs were infected with Ad-DN, generally in a dose-dependent manner. Thus, the mitogenic activity of DN-PPAR*γ* is likely mediated through these cell cycle regulatory proteins. Serum stimulation of Ad-DN-infected CASMCs further upregulated these proteins, while serum-induced increases in these proteins were substantially inhibited by Ad-WT or Ad-CA. Our in vitro results are consistent with recent reports that DN-PPAR*γ* represses the antiproliferative effects of WT-PPAR*γ* and increases intima formation in rat and mouse arteries and promotes VSMC proliferation and migration [17, 18]. Lim et al. [17] have also reported that DN-PPAR*γ* upregulates the expression of c-fos, an important component of the MAPK mitogenic signaling pathway. 

 These data further help to explain important functions of PPAR*γ* in the vasculature, which have been defined in mouse models which harbor a vascular-specific knockout of the nuclear receptor. Recently, Halabi et al. [14] developed a transgenic mouse with VSMC-specific expression of a DN-PPAR*γ* LBD mutant similar to the L486A/E471A PPAR*γ* LBD mutant that we describe in this report. This mouse developed hypertension, impaired nitric oxide-mediated vasodilation, an enhanced endothelin vasoconstriction response, and vascular hypertrophy with altered arteriolar remodeling. This mouse also demonstrated a robust increase in vascular osteopontin, an adhesion molecule that we have demonstrated plays a key role in accelerated atherosclerosis, and whose expression can be suppressed by PPAR*γ* ligand treatment [25]. Mice with cre/flox-generated endothelial- or VSMC-specific PPAR*γ* deficiency have also been shown to develop hypertension, although no histology was presented for these models and their vasodilator and vasoconstrictor responses were opposite to those reported for the VSMC DN-PPAR*γ* mouse model [26]. Both of the cre/flox models also demonstrated a blunting of diurnal variation in blood pressure and heart rate [26]. Recently, Chang et al. demonstrated that VSMC-specific PPAR*γ* KO mice exhibit impaired vasoactivity and hypotension which correlated with enhanced beta-2-adrenergic activity [27]. Taken together, these studies suggest that PPAR*γ* has important effects on vascular function, including regulation of cardiovascular rhythms, blood pressure, and VSMC proliferation, underscoring the importance of defining the mechanisms mediating these actions. 

CDK inhibitors, such as p21 and in particular, p27, are generally regarded as potent negative regulators in the cascade of G1 events induced by growth factors. In the present study, both p21 and p27 expressions were upregulated by DN-PPAR*γ*, although to a lesser degree than positive cell cycle regulators. CDKIs can prevent quiescent cells from entering cell cycle by inhibiting CDK activity and preventing Rb phosphorylation [28]. In this study, however, we found that DN-PPAR*γ* expression induced robust phosphorylation of Rb even in the presence of high levels of p27 and p21 and that the end result favored cell cycle progression. Data emerging from recent studies suggest that CDKIs p21 and p27 can also play a positive role in events during the G1 phase. Both CDKIs are required for cyclin D assembly with CDK4 and its stability and nuclear localization [29], and p27 levels were significantly associated with cyclin D1 and E expression in some breast cancer cell lines [30, 31]. We have also reported that PPAR*γ* ligands inhibit Rb phosphorylation and G1→S transition in rat aortic VSMC [6]. In that study, we found that PDGF plus insulin induced VSMC p21 expression and enhanced p27 degradation, while PPAR*γ* activation attenuated mitogen-induced p21 expression, perhaps contributing to the G1 arrest of VSMC in response to PPAR*γ* ligands. Hence, the prevailing view of CDKIs as universal inhibitors of CDKs may portray too simple a picture of their regulatory effects on the cell cycle [29]. 

The Ras/Raf/MEK/ERK signaling cascade appears to be indispensable for cell proliferation in a variety of different cell types [3]. ERK regulates the production of materials required for cell growth, including pyrimidine and ribosome synthesis [3], and transient expression of ERK1 antisense RNA or a kinase-deficient ERK1 mutant has been shown to decrease cell growth [32]. ERK activation also plays a key role in G1→S phase transition. Formation of cyclin D-CDK4/6 complexes is a key step for quiescent cells to enter the cell cycle, and activation of the ERK pathway is known to increase cyclin D1 expression, while inhibition of ERK activity by expression of a dominant negative form of MEK has been shown to decrease cyclin D expression [33]. We previously reported that the PPAR*γ* ligand troglitazone inhibited mitogen-induced MAPK signaling downstream of ERK phosphorylation and activation in VSMC, which was associated with a suppression of growth and ERK MAPK-controlled expression of MCM6 and MCM7, which are central components of a DNA replication complex [7, 34]. We also found that both PPAR*γ* ligands and the ERK specific-inhibitor PD98059 inhibited mitogen-induced p27 degradation and cyclin D1 up-regulation, contributing to the delayed G1→S transition of these cells [6]. In the present study, we found that DN-PPAR*γ* expression potently activated Rb and ERK phosphorylation and cyclin D1 expression, all of which were attenuated by pretreatment with PD98059. Taken together, these data strongly suggest that DN-PPAR*γ* regulates CASMC proliferation, at least in part, by inducing ERK 1/2 phosphorylation to activate cyclin D1 expression, and that the resulting cyclin D1-CDK4/6 complexes in turn phosphorylate Rb to promote G1→S progression.

Further studies are necessary to define the exact mechanism by which DN-PPAR*γ* increases ERK MAPK activity. PPAR*γ* may be active under basal conditions, in the absence of pharmacologic ligands, due to the presence of endogenous ligands such as oxidized fatty acids, which we have recently shown to reside in the large binding pocket of PPAR*γ* and to activate it in a biologically relevant manner (unpublished data). We found that medium chain C8-C10 fatty acids produced by the bacteria from which the crystallized nuclear receptor was cloned, and which are abundant in many foods, could stabilize Helix 12 of PPAR*γ* into an active conformation, as demonstrated by its ability to promote adipocyte differentiation, a well-known function of PPAR*γ*. DN-PPAR*γ* expression might therefore block basal CASMC PPAR*γ* activity, induced by endogenous ligands, which might otherwise suppress the ERK MAPK pathway. Gurnell et al. [13] previously demonstrated an impressive ability of this double mutant DN-PPAR*γ* construct to silence basal transcriptional activity, which was associated with increased corepressor association. We found basal PPAR*γ* activity to be about 30% of the activity obtained upon addition of pharmacologic ligand (Figure 1), similar to the 25% reported by Gurnell et al. [13]. Thus, it appears likely that these cells contain endogenous PPAR*γ* ligands, which may suppress cell growth in a PPAR*γ*-specific manner. This suggestion is supported by our observation that overexpression of WT-PPAR*γ* in the absence of synthetic ligand specifically suppresses positive cell cycle regulatory protein expression (Figure 4), while DN-PPAR*γ* expression does not alter cell cycle regulators in the absence of PPAR*γ* (Figure 6). Stimulation by FBS and DN-PPAR*γ* differed only in their effect on p27 and p21, which were decreased by the former and increased by the latter, but the cause and impact of these differences remains unclear. Surprisingly, DN-PPAR*γ*-stimulated ERK1/2 phosphorylation in serum-starved CASMC was equivalent to that induced by 5% FBS and lead to similar changes in cell cycle regulators, suggesting that endogenous PPAR*γ* ligands may have a substantial growth suppressive effect. Interestingly, Meredith et al. also observed a modest induction of mitogen-independent ERK activation in serum-starved VSMCs derived from mice expressing DN-PPAR*γ* (P465L), compared to VSMCs from mice expressing only wild-type PPAR*γ* [18]. The more robust effect on mitogen-independent ERK activation we observe after adenoviral overexpression of DN-PPAR*γ* may be due to the much higher levels of expression achievable through our in vitro approach. Meredith et al. found that DN-PPAR*γ*-expressing VSMCs exhibited enhanced proliferation and migration and they suggested that this effect could be mediated, at least in part, through Ets-1, which we have previously shown to be regulated by ERK signaling in VSMCs [35]. 

Excessive growth of VSMCs contributes to the formation of atherosclerotic and restenotic lesions. However, the growth-promoting capacity of DN-PPAR*γ* could prove beneficial in several gene therapy settings where reactivation of a dormant cell cycle would be desirable. For instance, proliferation of adult pancreatic islet cells is limited [36], and increasing their growth capacity could be beneficial for treatment of type I and II diabetes. The heart has little, if any, potential for regeneration after injury, since adult cardiac myocytes are terminally differentiated cells that do not proliferate. Many studies have attempted to induce cardiocmyocyte proliferation by overexpressing cell cycle regulatory molecules to promote cell cycle progression [37]. The potential for DN-PPAR*γ*, or compounds that mimic its effects, to promote reactivation of the cell cycle in these settings merits further exploration.

## Abbreviations

PPAR*γ*: Peroxisome proliferator-activated receptor *γ*
WT: Wild-type
DN: Dominant-negative
CA: Constitutively-active
CASMCs: Coronary artery smooth muscle cells
Rb: Retinoblastoma protein
MCM7: Minichromosome maintenance protein 7
ERK: Extracellular signal-regulated kinase
VSMCs: Vascular smooth muscle cells (VSMCs)
TZDs: Thiazolidinediones
LBD: Ligand-binding domain
IMR: Intima-media ratio
PPRE: PPAR Response Element
RSG: Rosiglitazone
CDKIs: Cyclin-dependent kinase inhibitors.
